# Identification of urban regions’ functions in Chengdu, China, based on vehicle trajectory data

**DOI:** 10.1371/journal.pone.0215656

**Published:** 2019-04-29

**Authors:** Qingke Gao, Jianhong Fu, Yang Yu, Xuehua Tang

**Affiliations:** 1 School of Remote Sensing and Information Engineering, Wuhan University, Wuhan, China; 2 Research Institute of Wuhan University in Shenzhen, Shenzhen, China; University of Sydney, AUSTRALIA

## Abstract

Data about human trajectories has been widely used to study urban regions that are attractive to researchers and are considered to be hotspots. It is difficult, however, to quantify the function of urban regions based on the varieties of human behavior. In this research, we developed a clustering method to help discover the specific functions that exist within urban regions. This method applies the Gaussian Mixture Model (GMM) to classify regions’ inflow and trip count characteristics. It regroups these urban regions using the Pearson Correlation Coefficient (PCC) clustering method based on those typical characteristics. Using a large amount of vehicle trajectory data (approximately 1,500,000 data points) in the Chinese city of Chengdu, we demonstrate that the method can discriminate between urban functional regions, by comparing the proportion of surface objects within each region. This research shows that vehicle trajectory data in different functional urban regions possesses different time-series curves, while similar types of functional regions can be identified by these curves. Compared with remote sensing images and other statistical methods which can provide only static results, our research can provide a timely and effective approach to determine an urban region’s functions.

## 1. Introduction

Cities comprise various functional regions. These regions include residential, educational, commercial, industrial, leisure zones, etc. [1]. Functional urban regions have been long noted as an important influence on how we recall, describe, and manage urban regions [2]. With the continuous progress of urbanization, urban areas constantly expand, and the types of functional urban regions became different from what was envisioned in early planning [3]. Understanding the changes in functional urban regions is critical for effective urban development planning, natural resource allocation, and ecosystem management [4]. In order to make better urban plans, it is important for planners to quickly and accurately identify different functional regions and understand their spatial structure within the city [3].

Remote sensing techniques have been an essential method for classifying urban land use, and are widely used to monitor changes in urban land. High resolution and hyperspectral remote sensing images can precisely identify urban land use type [5–9]. Most remote-sensing image-based land-use classification methods, however, focus on physical characteristics [4]. Thus, they are of little use in reflecting a region’s social characteristics. Data about points of interest (POI) is also widely used in urban land-use classification. Compared with other datasets and methods used in remote sensing, using POI data can lead to a better understanding of urban space and economic distribution at a fine-grained spatial resolution [10]. There is an identical problem, however, in that POI data is of no help in identifying social characteristics. Thinking in terms of functional urban regions essentially creates functional definitions of both economic activity as well as a city’s social”reach” [2]. Defining the functional identity of urban regions needs to consider human flow and trip count which reflect the region’s social characteristics. In order to solve this problem, location-based data is becoming increasingly popular and is used in this latest study. This data has been widely applied in human mobility pattern research. Many studies have used the data which can extract human locations to discover and compare in spatial terms the temporal patterns between urban regions.

Some studies focus on mobile phone data. Ratti first used mobile phone data with locations in Milan, Italy. The study sought to represent the intensity of urban activities and their evolution through space and time and discusses their future application and potential for urban studies and planning [11]. Niu explored the urban spatial structure through mobile phone positioning data in Shanghai using kernel-density analysis, identified different areas, and measured the degree of functional mix [12]. It has been attempted and found that the spatial-temporal sequence based on mobile phone call records can be used to classify urban plots effectively [13]. Some studies applied social media data. Yin used geo-tagged photos acquired from Flickr to analyze the distribution of some geographical topics in the USA by using the Latent Geographical Topic Analysis (LGTA) model. Yin found that the topics provided important cues to make it possible to group different geographical areas [14]. Lee analyzed the changing patterns of geographic regularity with time using data from Twitter and clustered urban types by tracking common patterns among the regions [15]. As for the trajectory data, Brockman used travel bugs to understand human mobility patterns [16]. Liu analyzed the globally spatial temporal pattern of trips and explored urban land use with GPS-enabled taxi data [17–20]. Yuan discovered different functional regions in Beijing city by combing the real transportation networks and the taxi data through a framework titled DRoF, which will help people easily understand a complex metropolitan area, benefiting a variety of applications [21].

The studies discussed above mainly focused on large-scale examinations of urban structure, such as a city’s center and sub-center, traffic source-sink areas, etc. In this study, we tried to identify a medium-scale of urban activity. Vehicle trajectory data and POI datasets are used to explore Chengdu’s functional urban regions. The contributions that this research makes are as follows:

1. We proposed a GMM-based method to extract information on functional urban regions based on human inflow and trip count characteristics in each region.
2. We applied the PCC clustering method to classify urban regions’ main time-series curve of inflow and trip count characteristics.
3. The proposed method can distinguish an urban region’s spatial temporal pattern, and identify similar functional urban regions, combined with POI analysis.

The remainder of this article is structured as follows. Section 2 introduces the study area and data preparation. Section 3 discusses the methods used in urban region classification. Section 4 introduces the results of our proposed method, and analyzes the regions’ main functions with the POI dataset. Section 5 presents our conclusions and points out the directions for our future work.

## 2. Study area and data preparation

The study area for this research locates is the Chinese city of Chengdu. As the capital of Sichuan province, Chengdu is located in southwest China. It has an area of 14600 square kilometers and has a population of approximately 16 million as of the end of 2017. In addition, Chengdu contains many ethnic groups and has residents from 55 ethnic minority groups. It comprises 11 administrative districts. Chengdu is a commercial logistics center and a comprehensive transportation hub. Its gross domestic product (GDP) exceeded 1300 billion yuan in 2017 and increased by 8.1% in that year compared to 2016. In 2017, there were 4,942,000 motor vehicles, and privately owned vehicles numbered more than 3,982,000. The vehicle trajectories can reflect the daily travel patterns of its residents based on the large amount of traffic. Because citizens mainly travel within the Fourth Ring Road area in Chengdu, we selected the districts within this area as the study area for this research.

Vehicle trajectory data in this study was collected by Didi Chuxing in Chengdu, China, which is published through its GAIA initiative (https://outreach.didichuxing.com/research/opendata/en/). The personal information contains in this data has been irreversibly processed anonymously. DIDI Chuxing provides a car-hailing service similar to Uber. People can order taxis or privately owned vehicles from this APP. This study used one week’s data, which recorded car-hailing order information from November 7 to November 13 in 2016. There are a total of 1.5 million records that were used for the entire study, ordered by date. This dataset covers a large proportion of the population from throughout Chengdu. A large number of studies indicate that human travel activities exhibit significant regularity and periodicity. Although this study only selects data within a one-week period, it still can reflect the spatial temporal patterns of Chengdu’s residents. The format of this dataset is shown in Table 1. Each record contains the location and timestamp of vehicle trajectory data and indicates where and when passengers embarked and debarked from vehicles. The ‘uid’ represents the unique identification of order data shown in Table 1.

**Table 1:** The order information of vehicle trajectory data.

Uid	On_time	Off_time	On_longitude	On_latitude	Off_longitude	Off_latitude
HxAaFlv0nlv0nvGAN5Inu9qdG9qwCuL8	1478450628	1478451078	104.072994	30.696191	104.064147	30.685848
NBH4HsH6nrvcpvMHD7Aiw9ljv3huByH2	1478452703	1478453830	104.066220	30.690230	104.027710	30.631710
…	…		…	…		…
JzI8GlH6qiJ2oqEIN@xru9ekw3iqFHD8	1478500417	1478501587	104.076580	30.621710	104.077220	30.664740
EuC_FtA5roJ5hnJIGbGsE_fdw9nzNBLa	1478479814	1478480639	104.068728	30.657195	104.042400	30.660080
…	…		…	…		…

The Open Street Map (OSM) is a collaborative project to create a free editable map database of the world as is probably the most well-known example of Volunteered Geographic Information [22]. As shown in Table 2, the road network dataset was downloaded from the OSM website, which lists 10,452 roads. There are, however, several special features in the road network, such as overpass bridges, traffic circles (roundabouts), exit ramps, etc., which need to be eliminated in the network preprocessing. We can therefore identify them by attributes such as “one way,”“bridge,” and “tunnel.” An attribute of ‘”class” is the types of roads. Each road is different, but we selected only some main roads, from a larger array that included some high-level roads such as highway to low-level roads such as foot paths.

**Table 2:** The road network data of OSM.

osm_id	code	fclass	name	oneway	maxspeed	layer	bridge	tunnel
99989683	5113	primary	Shawan Road	F	0	0	F	F
99989684	5113	primary	Tongjinqiao Road	F	0	0	F	F
…	…	…	…	…	…	…	…	…
347928103	5122	residential	Renhou Street	B	0	0	F	F
281244893	5115	tertiary	Gongxing Road	B	0	0	F	F
…	…	…	…	…	…	…	…	…

In this study, the POI dataset was fetched via application programming interfaces (APIs) provided by Baidu Map Services (http://map.baidu.com), which is the most widely used search engine and map service provider in China [23–24]. There is a total of 541,047 POIs inside Chengdu’s Fourth Ring Road. The detailed format of original POI data is given in Table 3. Several attributes of each POI are available in the dataset. The attributes include name, longitude, latitude, address, telephone, and type. There are 16 types of POIs as shown in Table 4. We list the relevant detail of each type which can reflect their main composition.

**Table 3:** The POI data of Chengdu.

Name	Longitude	Latitude	Address	Type
Dream color hotel	104.000063	30.663917	No.118 rui nam street	Accommodation
Alibaba cloud computing co	104.073704	30.63071	No.9, section 4, renmin south road	Business
…	…	…	…	…
Kelly kitchen	103.959824	30.686032	144 rayline west road	Food
Bailianchi police station	104.139989	30.729701	Near 1323 panda avenue	Government
…	…	…	…	…

**Table 4:** The types of POIs data.

Category	Detail	Category	Detail
Accommodate	Hotel and guesthouse	Hospital	Hospitals
Resident	Community, residential building, dormitory, house	Leisure	Movie theater, entertainment, sports, vacation and leisure
Banks	Bank, insurance, securities and finance companies,	Vehicle	Car fix, car sale, car service, motor shops, hardware store, maintenance
Business	Companies, enterprises with agriculture, forestry, fishing	Industrial	Industrial park building, Industrial buildings
Nature	Scenic spots, park plazas, natural scenery	Public	Public toilets, newsstands, shelters
Food	Teahouse, cafe, fast food restaurant, ice cream shop, dessert shop, Chinese and western restaurant	Shopping	Convenience stores, supermarkets, shopping malls, specialty stores, personal items, home appliances and other stores
Culture	Museums, archives, exhibition centers, Schools, science and technology museums, art galleries, libraries, cultural palaces	Live	Electricity, communication, lottery, logistics business hall, bathhouse, barbershop, laundry, post office
Government	Government agencies, industry and commerce, public inspection laws organizations, social organizations	Transport	Bus station, subway station, railway station, parking lot, airport, long-distance passenger station

After data preparation, the original data needed to be preprocessed. That task includes data clearing and trip extraction for vehicle trajectory and POI data. Moreover, we needed to determine the scale of the urban regions in order to meet the experimental requirements. Changing grain size, extent, and the direction of analysis (or sampling) using several different unit scales will create different analysis results [25]. In studies that have been created on this topic, regular grid networks, such as squares of 1km x 1km, were usually applied as the base map for statistical purposes. A uniform grid, however, clearly differs from the streets and blocks in the real world, making it difficult to find what scale of regular grid should be used. The larger the grids, the greater the mix of functional urban regions contained within that grid. Similarly, a smaller grid will result in little data or in missing internal data points within the square. Both results will make it impossible to identify the actual function of urban regions accurately.

**Fig 1:**
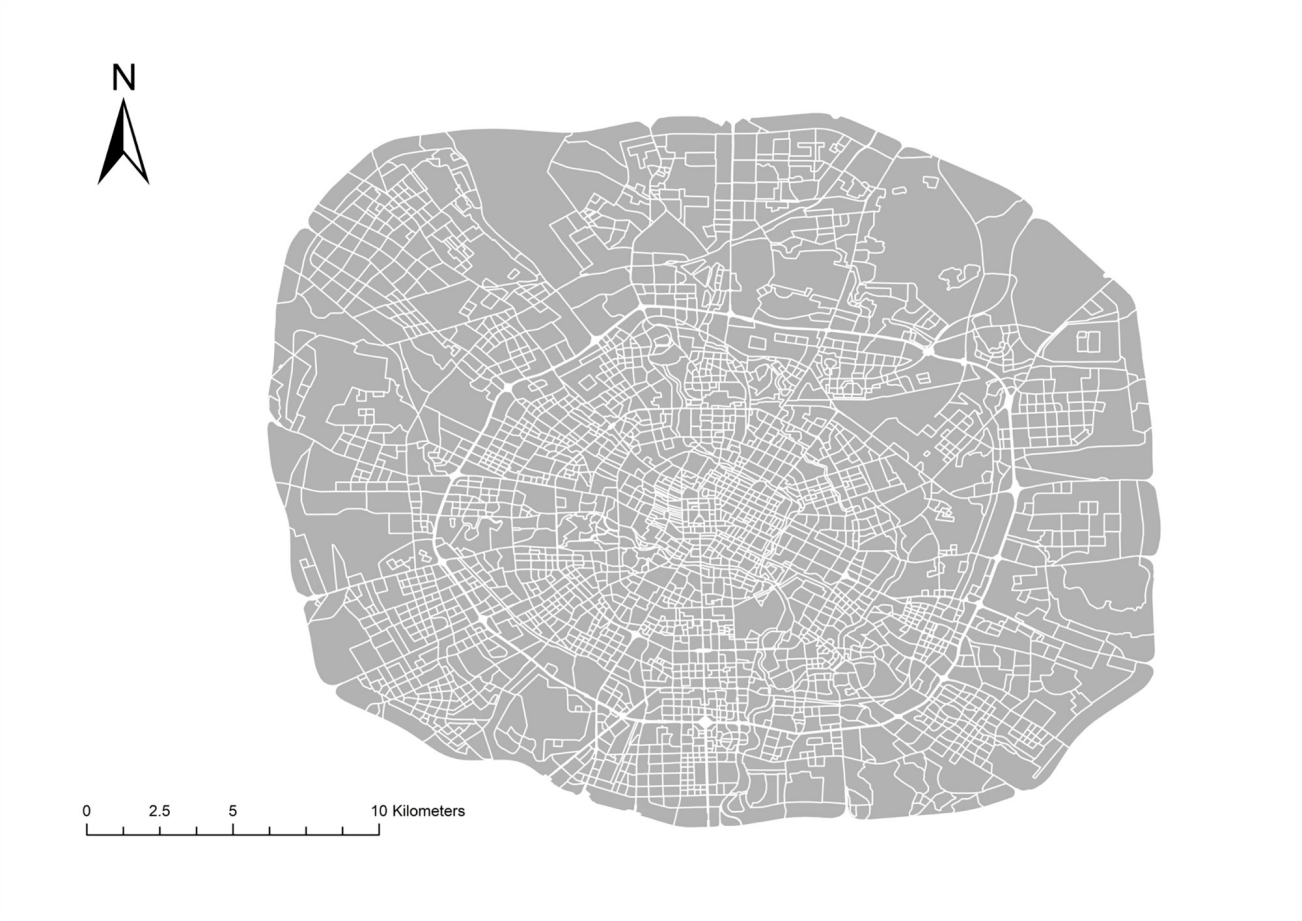
The urban regions after segmentation with road network.

Entering and leaving a vehicle usually occurs on the side of the road. Thus, all of the data points are distributed along roads. For this reason, this study used the actual road network to divide the urban area. Considering there are some the dual lines road such as a regional highway and a national road, we use the centerlines of such roads to divide urban regions. Data outside the study area were not included. In particular, some vehicle trajectory points distribute in the center of a road. To match these points with specific regions, this study also buffered the polygon to cover the spindly blank areas around the regions. Data processing results with the road network are shown in Fig 1. The urban area has been divided into many irregular regions with the road network as the line dividing those regions.

## 3. Method and methodology

### 3.1 Gaussian mixture model based region aggregation

Trip count is a useful characteristic to identify functional regions. From vehicle trajectory data, we can extract human flow by counting each region’s rate of pick-ups and drop-offs for people within a designated unit of time. Intuitively, we can use some simple methods, instead of the density calculation, such as counting the number of points in a region or a buffering area. These counting methods could raise bias, however, so it is recommended to use density to make a time series [26]. In this study, we divided the urban region into irregular regions. Crowd density was used as the main characteristic in order to be able to generalize. For each hour, for seven days, we computed the density of pick-up and drop-off points in the study area as shown below. The unit of area is in square kilometers.

$$DN(i)=\frac{\sum_{i}^{M}(\sum_{j}^{\beta_{x}}P_{\left(i,j\right)})}{S_{i}}{,\;\beta }_{x}\in N,\;x\in [1,168]$$ (1)

$$P_{\left(i,j\right)}=\left\{ \begin{array}{l} \;0\;j\;falls\;in\;block\;i\\ 1\;j\;falls\;outside\;block\;i \end{array}\right.$$ (2)

M is the collection of regions. N is the collection of pick-up/drop-off points at all times. *β*_*x*_ is a set of pick-up/drop-off points that belongs to N during the time period *x*. *S*_*i*_ is the area of region *i*. *P*_(*i*,*j*)_ indicates whether the j point falls in region *i*.

For further analysis, *DN*_*pick*−*on*_(*i*) represents the density of pick-up points in region *i*, and *DN*_*drop*−*off*_(*i*) represents the number of people getting off the bus per unit area of region *i*.

$$inflow(i)={DN}_{drop-off}(i)-{DN}_{pick-up}(i)$$ (3)

$$tripcounts(i)={DN}_{pick-up}(i){+DN}_{drop-off}(i)$$ (4)

*inflow*(*i*) represents the inflow density of region *i*, whose value changes with time, indicating the density of people flowing in from the region during each set time period. Its values can be positive or negative. Positive means inflow, and negative means outflow. In the same way, *tripcounts*(*i*) represents the trip count of region *i*. Both *inflow*(*i*) and *tripcounts*(*i*) can be thought of as a region’s time-series characteristics.

**Fig 2:**
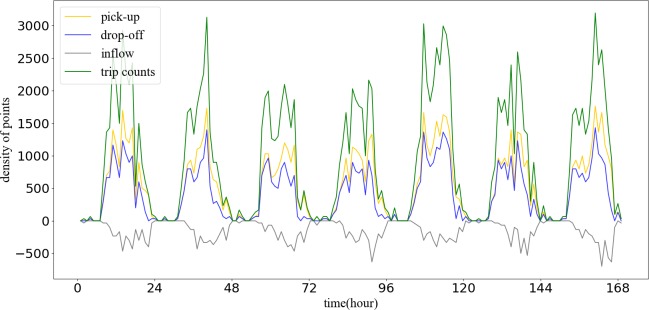
Sample of a region’s time-series characteristics.

According to the equations above, we can calculate the time-series characteristic curve based on the pick-up and drop-off points of different regions. Fig 2 shows a sample of more than four characteristics, which includes pick-up, drop-off, inflow, and trip count. It can easily determine that the inflow and trip count characteristics are more distinct than the pick-up and drop-off curve. Thus, these two characteristics are more useful to extract the region’s spatial patterns.

**Fig 3:**
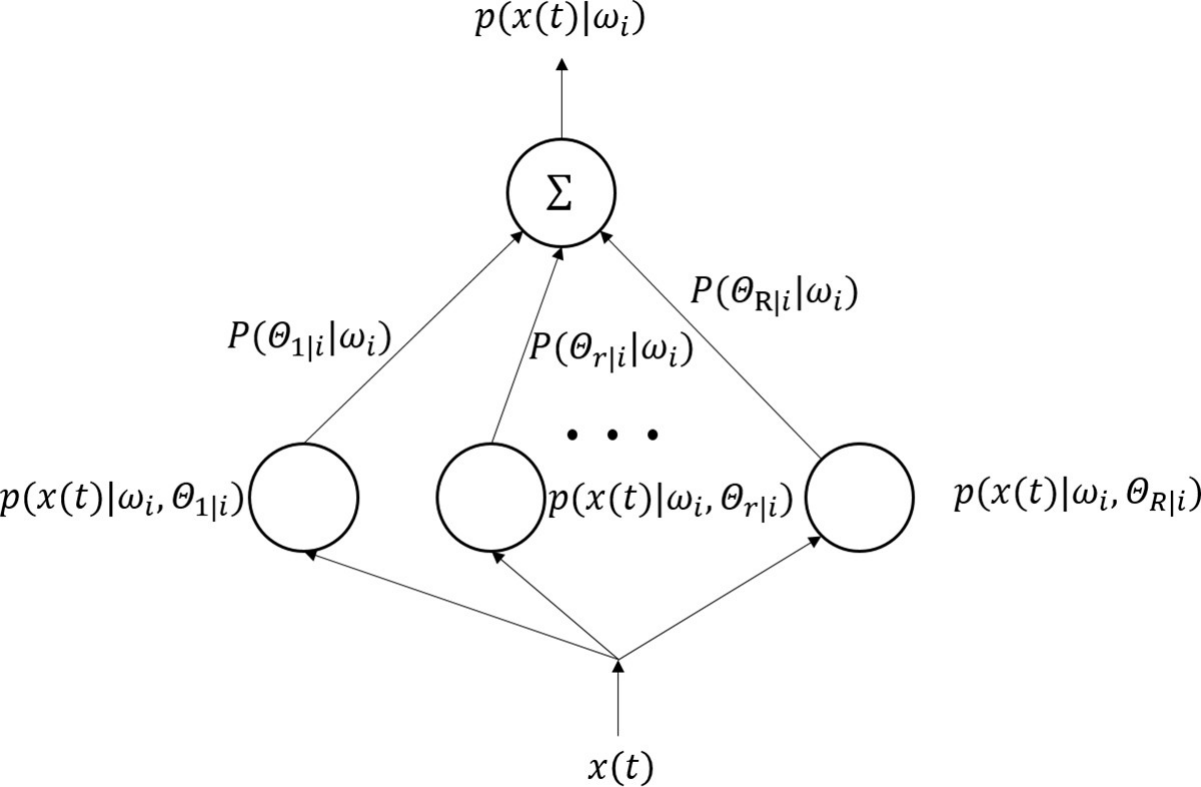
Architecture of a GMM.

We assume that many regions have similar time-series characteristics. Therefore, the next step is to separate these regions into different groups. Different clustering algorithms have different advantages and usage scenarios. The probability of each sample for each category will be calculated which will help us determine the region’s aggregation. Each sample is a part of a certain cluster. A part of a certain type of sample constitutes a distribution of that cluster. As a result, GMMs are widely used in data mining. They are a semi-parametric technique for estimating probability density functions [27]. This algorithm only maximizes the probability. It does not make the mean approach zero, nor does it make the clustering class appear as special structures that may or may not be applicable [28]. GMM can output data points belonging to a certain type of probability, so the output information is much richer. The output of a GMM is the weighted sum of R component densities, as shown in Fig 3.

Given a set of *N* independent and identically distributed patterns *χ*_*i*_ = {*x*(*t*);*t* = 1,2,3…,*N*} associated with class *ω*_*i*_, we assume that the class likelihood function *p*(*x*(*t*)|*ω*_*i*_) for class *ω*_*i*_ is a mixture of Gaussian distributions, i.e., the principal components F1 to F5 are independent and identically distributed patterns. We can put the each component into this formula as *x*(*t*) after dimensions reduction,

$$p(x(t)|\omega_{i})=\sum_{r=1}^{R}P(\Theta_{r|i}|\omega_{i})p(x(t)|\omega_{i},\Theta_{r|i})$$ (5)

where *Θ*_*r*|*i*_ represents the parameters of the *rth* mixture component, R is the total number of mixture components, *p*(*x*(*t*)|*ω*_*i*_,*Θ*_*r*|*i*_)≡*N*(*μ*_*r*|*i*_,∑_*r*|*i*_) is the probability density function of the *rth* component, and *p*(*x*(*t*)|*ω*_*i*_) is the prior probability (also called mixture coefficients) of the *rth* component. Typically, *N*(*μ*_*r*|*i*_,∑_*r*|*i*_) is a Gaussian distribution with mean *μ*_*r*|*i*_ and covariance ∑_*r*|*i*_.The training of GMMs can be formulated as a maximum likelihood problem where the mean vectors {*μ*_*r*|*i*_}, covariance matrices {∑_*r*|*i*_}, and mixture coefficients {*P*(*Θ*_*r*|*i*_|*ω*_*i*_)} are typically estimated by the EM algorithm.

The dataset of inflow and trip count after dimension reduction are independent and identically distributed patterns, so we can place them into the formula above, as *x* (*t*). Meanwhile, we will determine the number of *R* clusters by the Bayesian Information Criterion (BIC) calculation [29]. Thus we can calculate each cluster’s contribution *P*(*Θ*_*r*|*i*_|*ω*_*i*_) to the dataset and the probability density function *p*(*x*(*t*)|*ω*_*i*_,*Θ*_*r*|*i*_) in the *r*_*th*_ cluster. We then get the sum of the mixture of Gaussian distributions of the R cluster to get the *p*(*x*(*t*)|*ω*_*i*_). Finally, the probability of each sample for each cluster will be obtained which will help us for determining the region aggregation.

### 3.2 Urban region classification based on the Pearson correlation coefficient

After aggregation, regions will be separated into several clusters. Each cluster can calculate its average trip count and inflow density on different days. Sometimes the amount of human density is different in different places. In China, most people like to live near the city center so density may be affected by location. We can, however, assume that similar types of functional regions have similar trip count and inflow density curve shapes. It is clear that the inflow and trip count density curves are similar, and the distinction is based on the density of the regions.

We can regroup the clusters above after normalization. We assume that similar types of functional regions have higher relationships in inflow and trip count characteristics. PCC clustering can be used to discover these groups. The PCC score quantifies how well two data objects fit in a line. Unlike the Euclidean Distance similarity score, the score measures how highly correlated two variables are and is measured from -1 to +1. A PCC score of 1 indicates that the data objects are perfectly correlated. In this case, however, a score of -1 means that the data objects are not correlated [30]. In the mathematical form, the score of two data *x* and *y* can be described as:

$$Person(x,y)=\frac{\sum xy-\frac{\sum x\sum y}{N}}{\sqrt{\left(\sum x^{2}-\frac{{\left(\sum x\right)}^{2}}{N})(\sum y^{2}-\frac{{\left(\sum y\right)}^{2}}{N}\right)}}$$ (6)

*N* is total number of attributes. As a result, the PCC has been used by researchers to compare time-series data sets to assess the temporal similarities. The correlation coefficient can be used to evaluate the entire curve as opposed to discrete data points [31]. Our results after aggregation are some curves of clusters. Thus, we can use PCC to regroup the clusters through their density curve shape after data standardization.

## 4. Results and discussion

### 4.1 Region aggregation results

**Fig 4:**
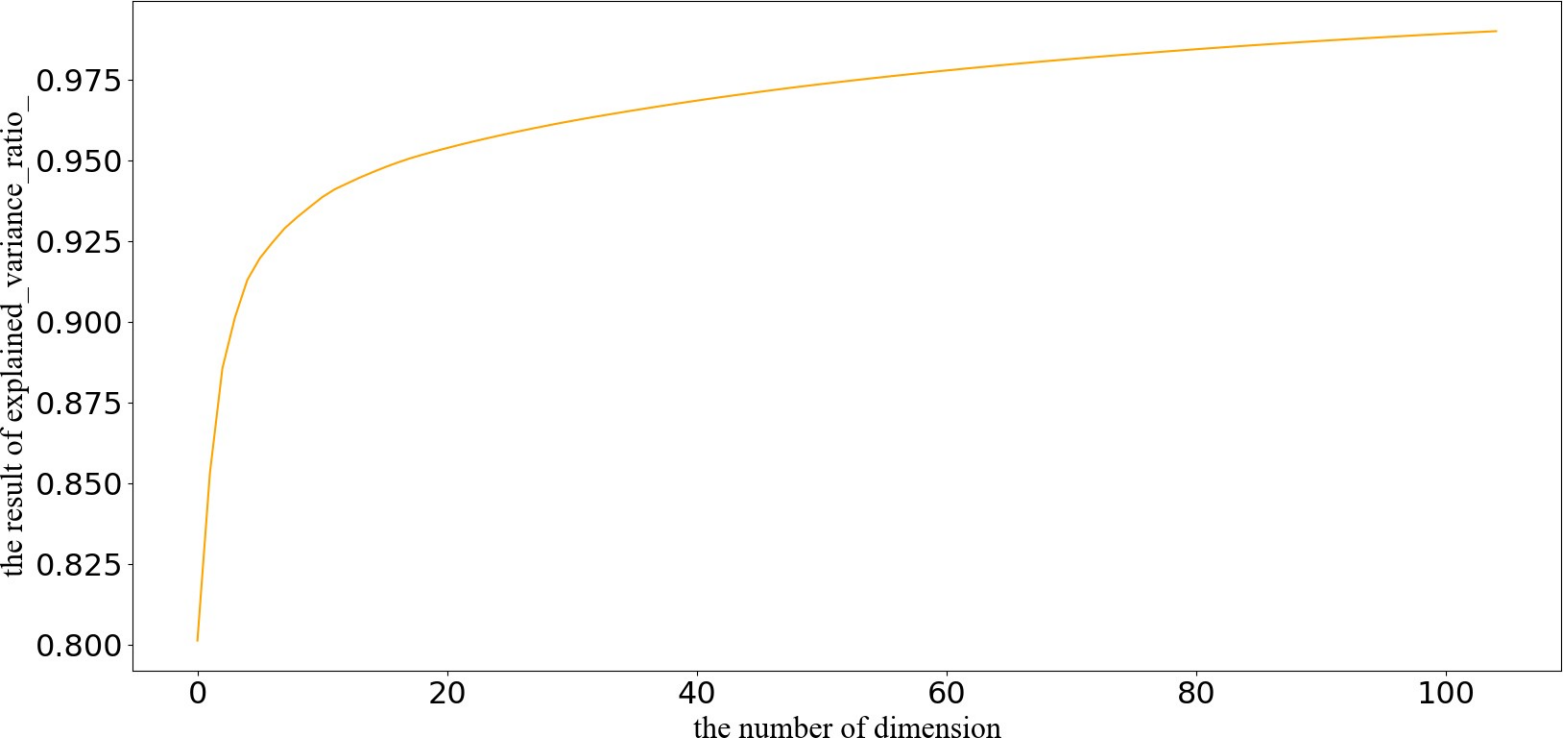
The cumulative distribution function of percentage of variance explained.

As described in Section 3, before running the GMM, we first need to define some parameters. When a dataset contains a large number of attributes open to inference attacks, we face a choice of either completely suppressing most of the data or of losing the desired level of anonymity [32]. This experiment used a week's worth of data, 24 hours a day, seven days a week. The total dimension of inflow and trip count characteristics is 336, which will be used in the next step. It must be taken into consideration that many dimensions are useless. For example, there are few pickups and drop offs of people between 12 am to 6 am. The Principal Component Analysis (PCA) can be applied to extract the main characteristics from this high-dimensional dataset [33]. The cumulative distribution function of the percentage of variance explained by each selected component is shown in Fig 4. We chose to keep 90% of the variance, with the result that we had five principal components. The dataset after dimension reduction would be used for region aggregation.

**Fig 5:**
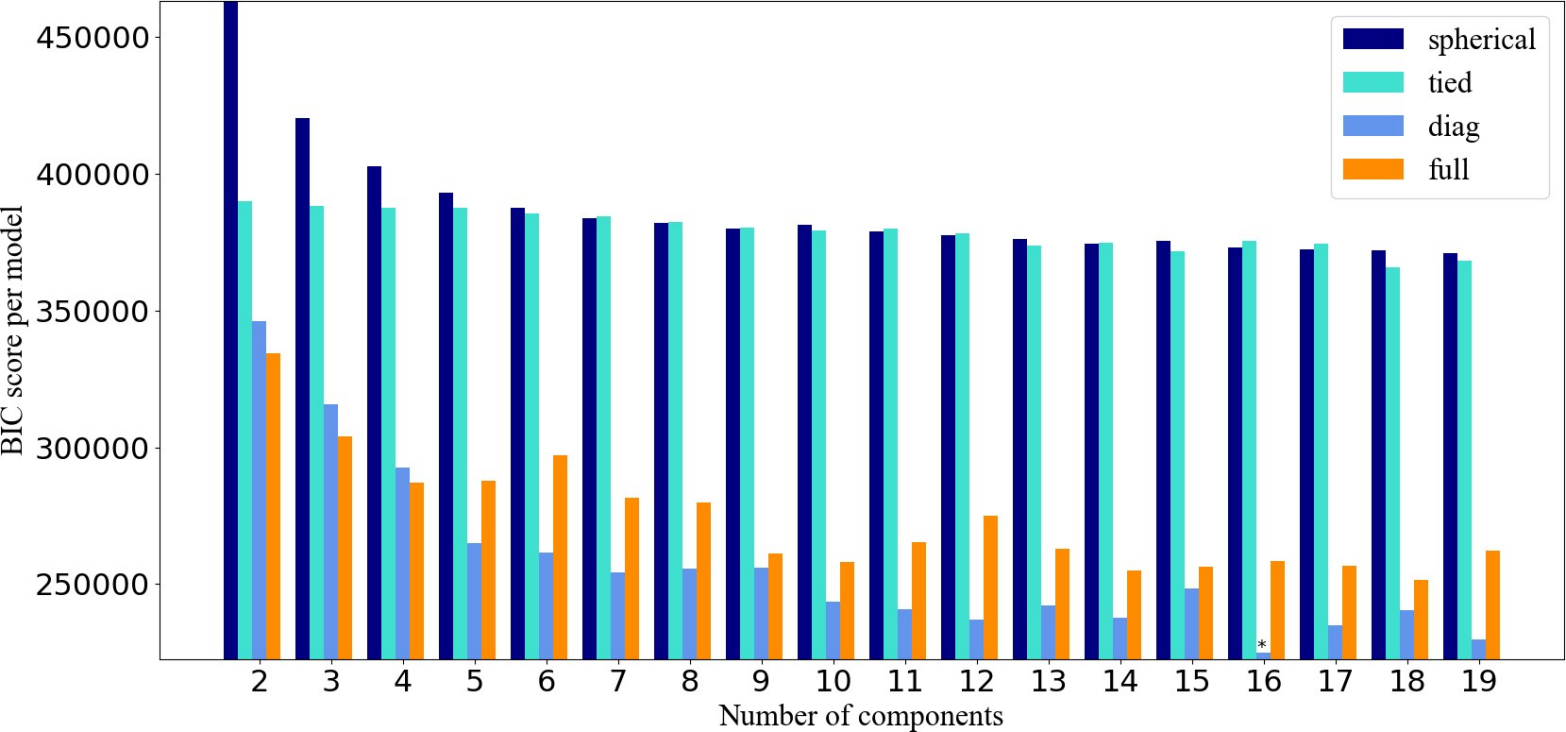
The BIC values for different covariance patterns and different components.

As a complex algorithm, GMM also has some attributes that must be determined before it is run. First, it is important to determine the number of original centers of clustering. Some researchers used the Bayesian Information Criterion (BIC) to find the fittest model to approximate the distribution of the instances [29]. BIC is a measure of the model's fitting degree and complexity. The larger the value, the worse the model's fit. The smaller the value, the better the model’s fit. Another parameter is the covariance of the different classes estimated method, such as spherical, diagonal, tied, or full covariance [28]. The covariance type in the GMM model controls the shape freedom of each cluster. To determine the most suitable covariance type, we calculated BIC values for different covariance patterns with different components. The results are shown in Fig 5.

**Fig 6:**
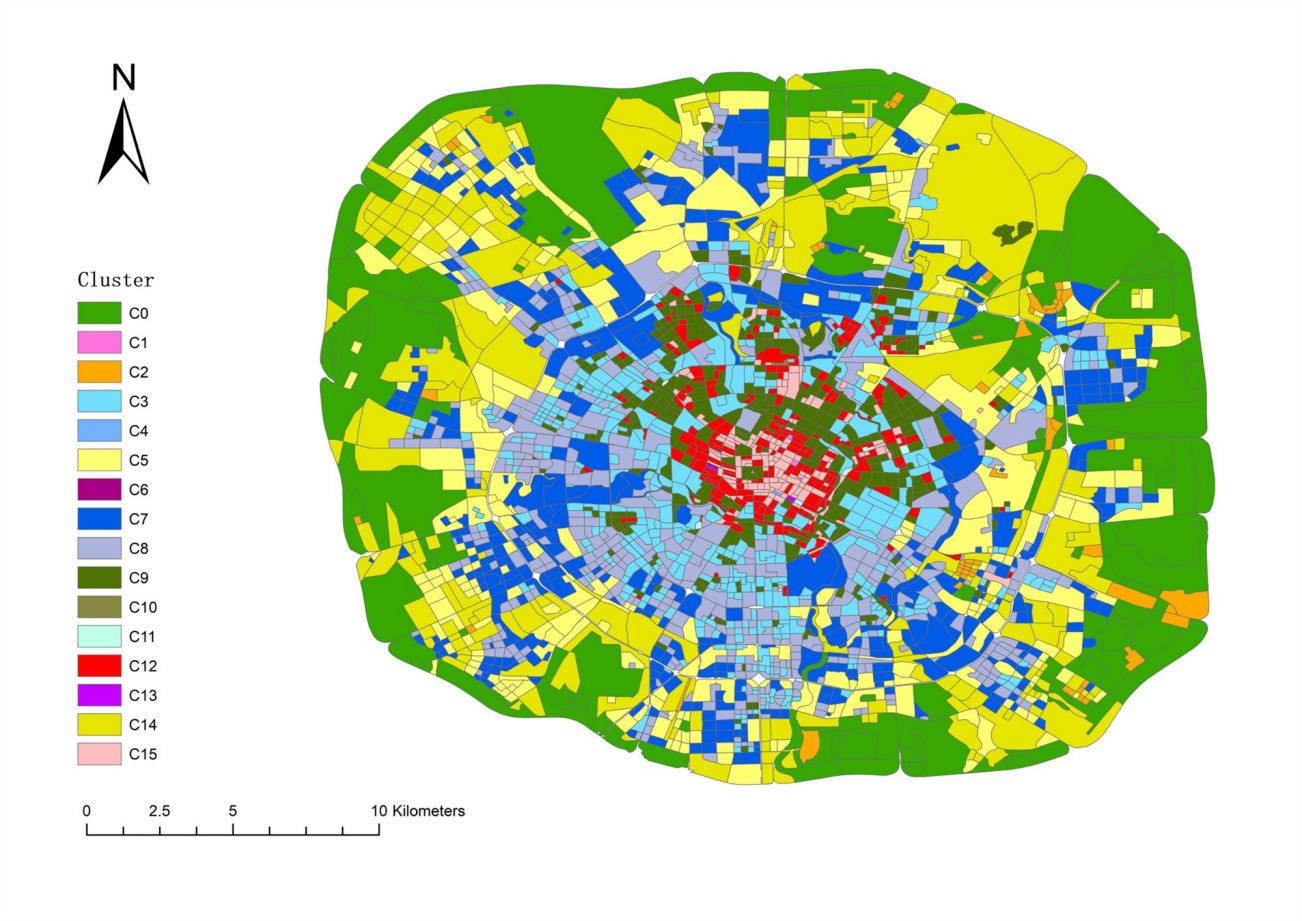
The spatial distribution of region clustering result.

In Fig 5, the horizontal axis represents the clusters, the different colors of the columns represent different covariance model of BIC scores. In this plot, "*" marks the location of the column where many of the best components are, as well as the best type of covariance BIC score. Finally, when the covariance model is "diag" and components equal 16, the BIC scored lowest. We obtained the vehicle trajectory clustering statistics by incorporating these parameters into the GMM clustering model, as shown in Table 5. The clusters are labeled from C0 to C15. Except for some clusters with a few counts, most remain above 100, and some nearly 500. The result is also mapped as shown in Fig 6.

**Table 5:** Urban region clustering statistics.

cluster	C0	C1	C2	C3	C4	C5	C6	C7	C8	C9	C10	C11	C12	C13	C14	C15
**count**	132	3	56	457	1	299	1	383	475	351	1	6	239	2	237	167

**Fig 7:**
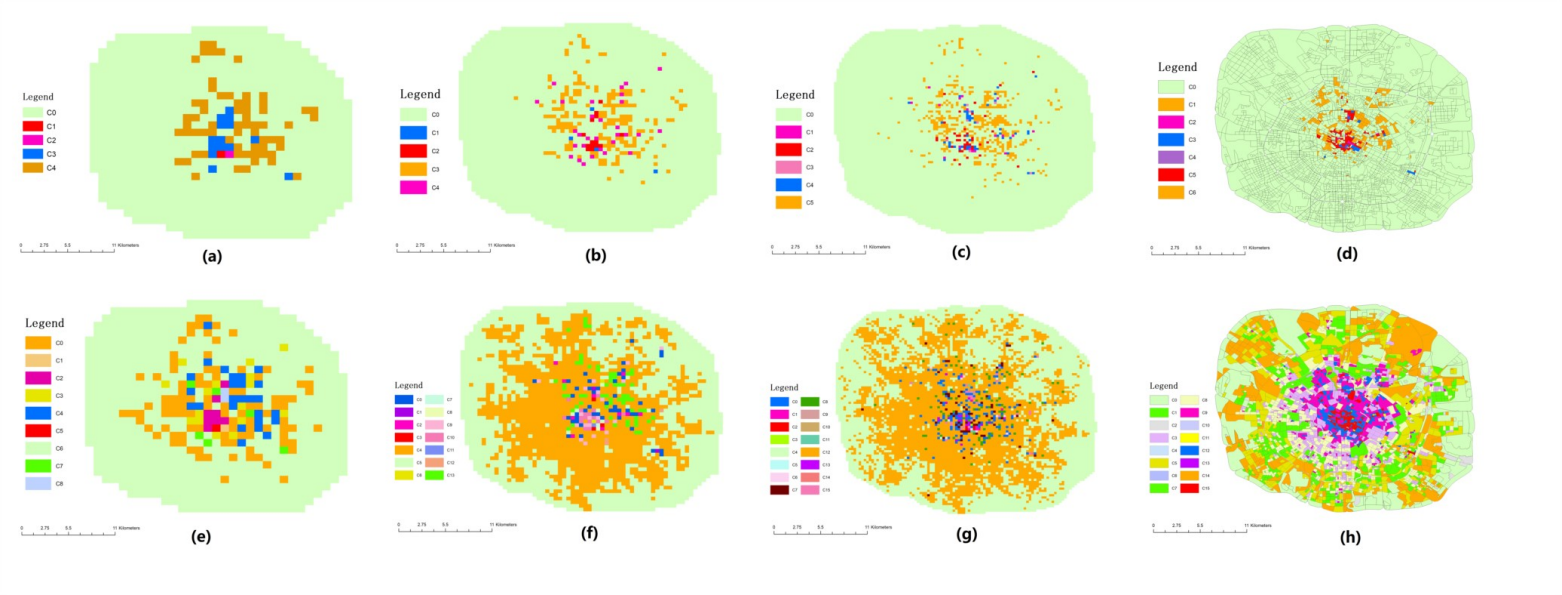
Compare the GMM-based region clustering algorithm with K-means algorithm: (a) K-means clustering with 1km×1km cells; (b) K-means clustering with 500m×500m cells; (c) K-means clustering with 300m×300m cells; (d) K-means clustering with road network segmentation; (e) GMM based clustering with 1km×1km cells; (f) GMM based clustering with 500m×500m cells; (g) GMM based clustering with 300m×300m cells; (h) GMM based clustering with road network segmentation.

In addition, we compare the GMM-based algorithm with a K-means algorithm, which is in the original research [19]. Different urban segmentation methods are also used to test the clustering results shown in Fig 7. It contains four types of division methods: (1)300m×300m cells; (2)500m×500m cells; (3)1km×1km cells; and (4) segmentation with road network. It shows the obvious conclusion that the divided urban areas are more well-distributed by the GMM-based method in all types. It can decrease the cell size to improve the clustering result, but another problem is that many empty value cell will be generated if the size is too small, a situation that can be found in Fig 7B and 7C. Furthermore, as is the case for both the grid and road network division methods, the different types of areas in suburban regions are still hard to distinguish by the use of K-means. It may imply that the GMM with the road network divide method can play a better role in performing a functional region analysis.

### 4.2 Cluster regroup results

**Fig 8:**
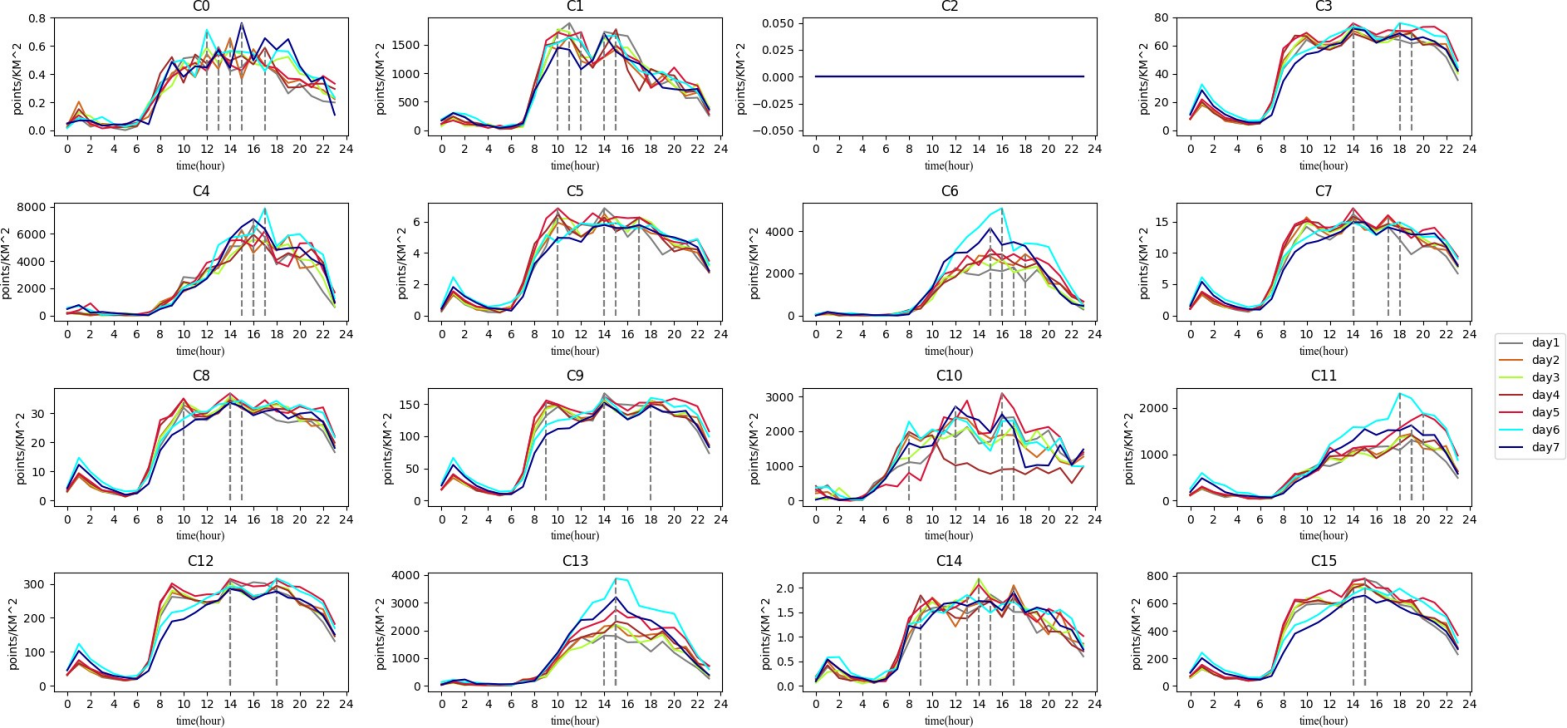
The trip count of clusters.

**Fig 9:**
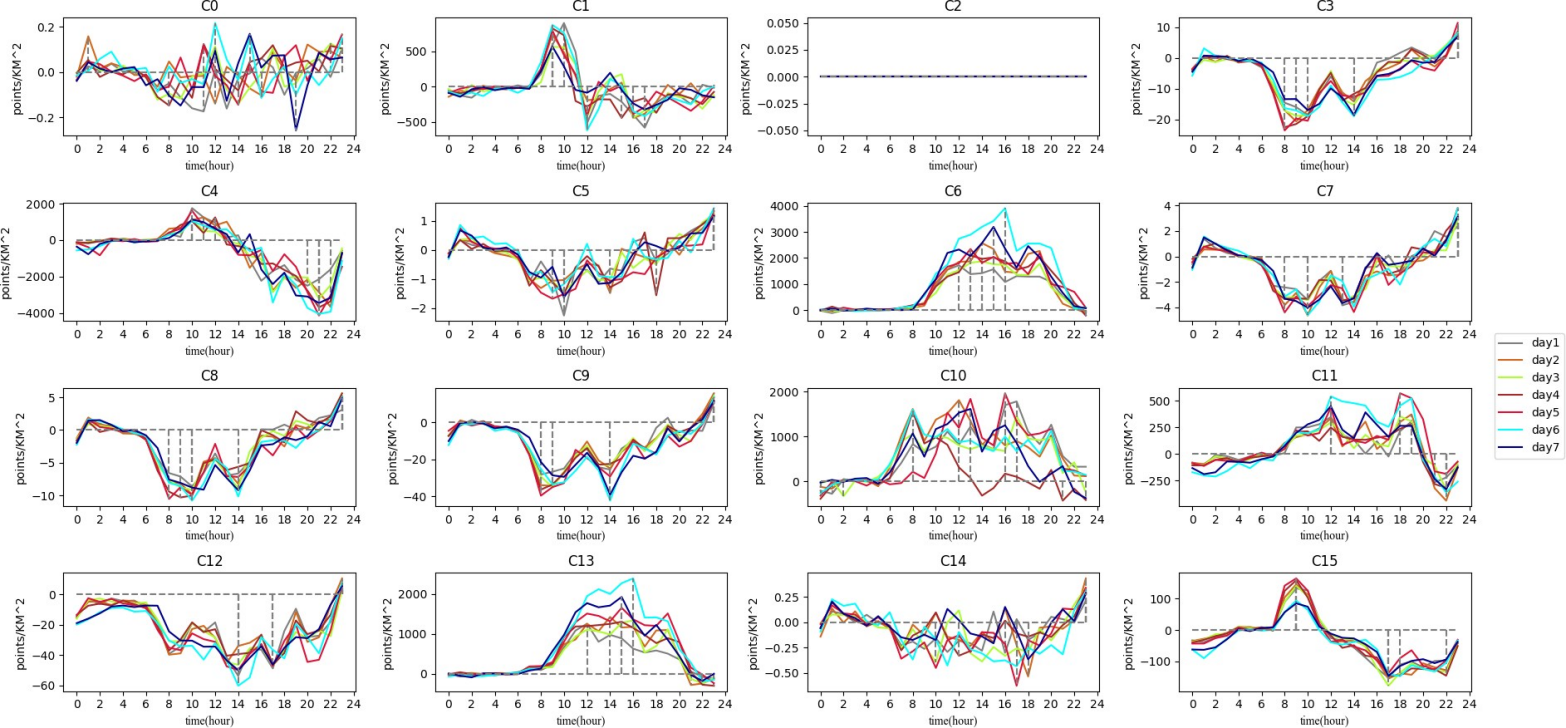
The inflow of clusters.

In order to analyze the clusters’ function, the average time curve of inflow and trip count characteristics belonging to each cluster is calculated as shown below. Fig 8 shows the mean trip count time curves of clusters. Fig 9 shows the mean inflow time curves of clusters.

In Fig 8, trip count among clusters are different. It is easy to see that C0 and C14 have small trip count. C4, C6, C10, C11, and C13 have a large vehicle trajectory flow. There is no data in C2. The crests and troughs of each wave are also different in both peak count and peak time. The inflow of each cluster also has some differences (Fig 9). Some clusters have positive inflow before 12 am (such as C1 and C15), while some have positive inflow all times (such as C6 and C13).

**Fig 10:**
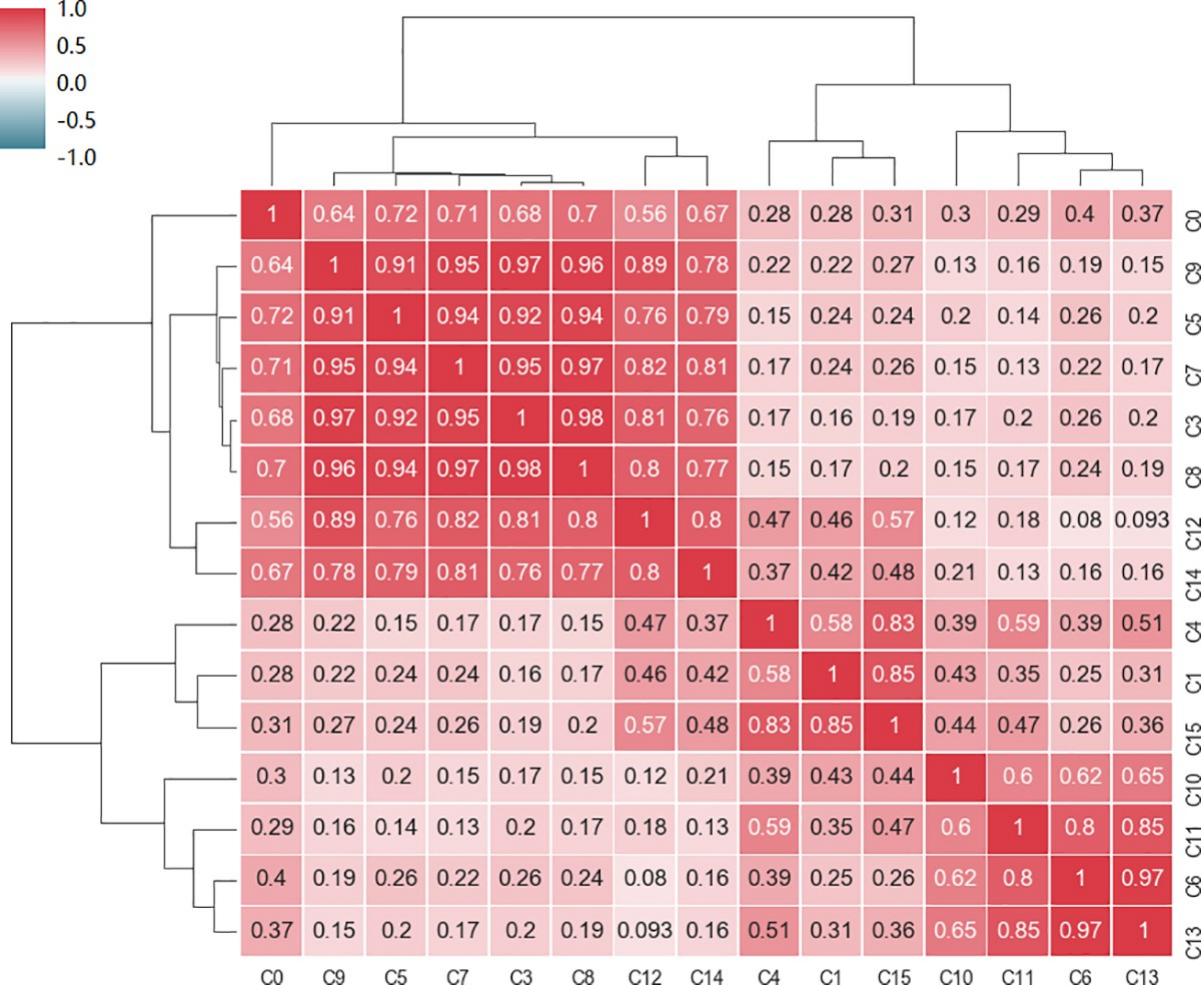
Correlation matrix of clusters.

Because a coefficient correlation of 0.8 is high enough in complex social science studies, this study used that value as the threshold. If the relationship between two clusters exceeds 0.8, they may have the same function. The C2 cluster has little vehicle trajectory data so we ignored it before regrouping. Using the PCC-based classification we discussed in Section 3.2, the results can be seen in Fig 10.

Based on the coefficient correlation, the clusters can be reclassified into seven groups. We labeled the groups as G1 through G7. Table 6 shows the groups and their subordinate clusters

**Table 6:** Regions classification results.

**Group**	**G1**	**G2**	**G3**	**G4**	**G5**	**G6**	**G7**
**Cluster**	C0	C3,C5,C7,C8,C9	C12,C14	C1,C15	C4	C10	C11,C6,C13

### 4.3 Urban region function analysis

In order to explore the variability of the seven groups, we further investigated their main function with POIs. People usually travel to different POIs for different kinds of activities. Therefore, different types of POIs reflect particular urban functions [10]. We propose that the density and ratio of POIs can be used to divide regions into different types. After deriving the clusters above, each cluster needs to have its main function confirmed with POIs. The same types of POIs can be located in different region types, but the same region types may have the similar, main POIs types most of the time [34]. For example, the resident regions’ main POIs are house and apartment while commercial regions’ main POIs are mall, market, and shop. In order to understand the functions within each group, we use the frequency average density (*FD*) and category ratio (*CR*) to identify it as below:

$${FD}_{ij}=\frac{n_{i}}{S_{j}}(i=1,2,\ldots ,k;j=1,2,\ldots ,N)$$ (7)

$${CR}_{ij}=\frac{{FD}_{ij}}{\sum_{i=1}^{k}{FD}_{ij}}(j=1,2,\ldots ,N)$$ (8)

**Fig 11:**
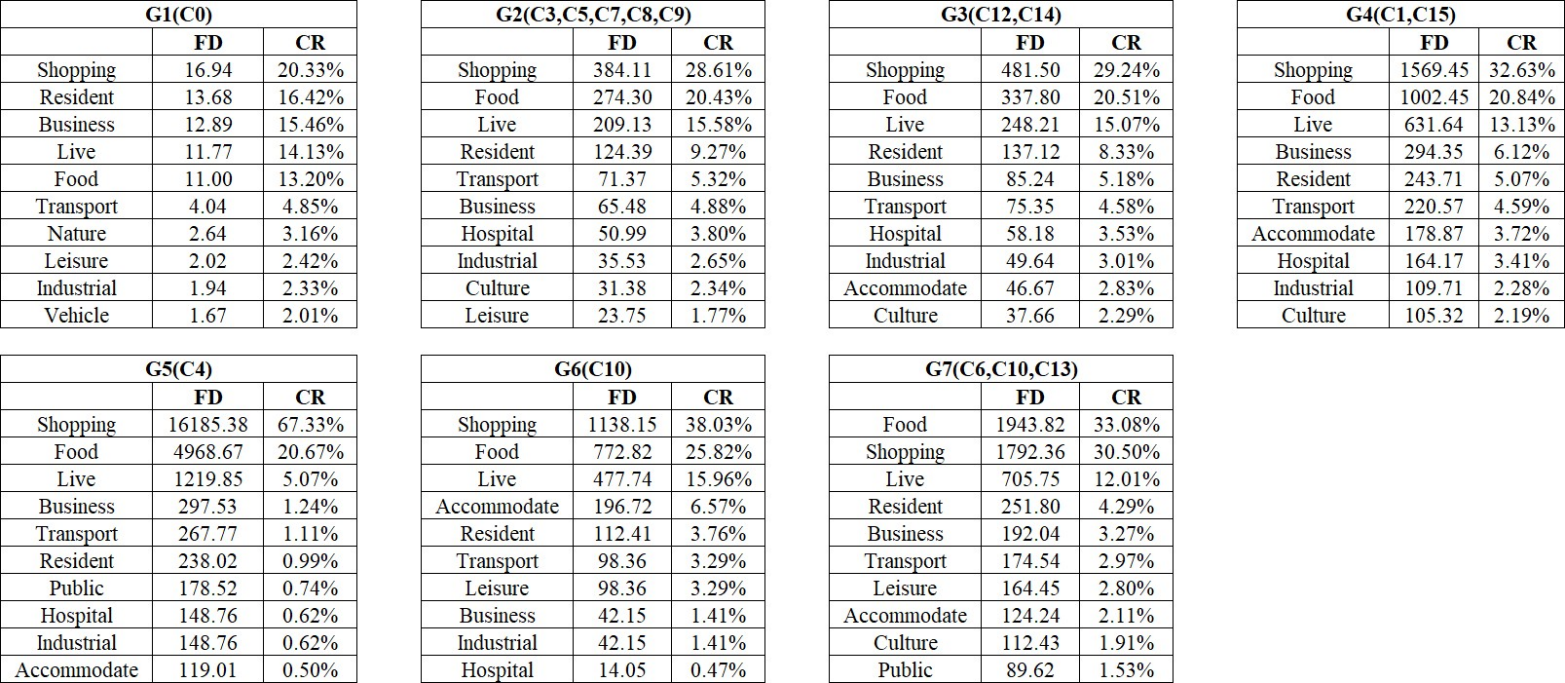
Top-10 ranked POIs types of urban region group.

We assume that POIs here can be separated into *k* types, *n*_*i*_ is each type of POI count in the region j. *S*_*j*_ means the area of region j. *FD*_*ij*_ represents the density of the *i* type of POIs in the j region. *CR*_*ij*_ represents each type of POI density ratio in the region j. We can use *CR*_*ij*_ to identify the group’s main function. As a result, we can calculate the mean *FD* and mean *CR* of each group which we aggregated before. In Fig 11, we show the top 10 ratios of each group.

**Fig 12:**
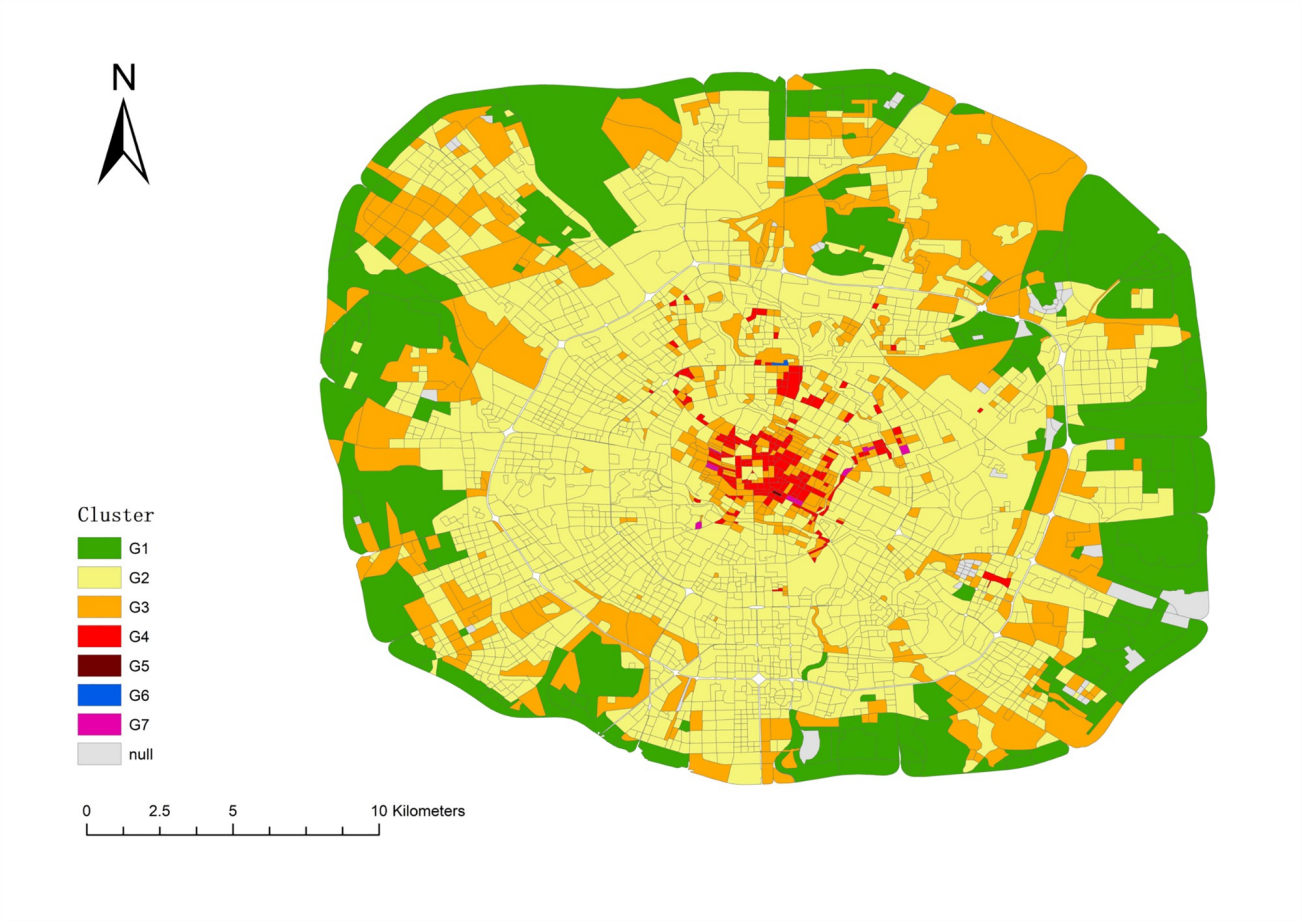
Distribution of regions classification results.

Chengdu is a state tourist city. Therefore, food and shopping POIs are obviously larger than other POIs. However, other types of POIs in a group can indicate the distinction as shown in Fig 11. To understand descriptive characteristics of the urban region function within each group, we computed the average of each cluster’s inflow and trip count. The results are elaborated in Fig 12. Based on Figs 11 and 12, we interpret the group function as follows.

Under G1, the density of each type of POI is the lowest among all groups which means the living population there is very small. From the map, we can see that G1 is distributed around the 4^th^ ring of Chengdu which is far from the center of city. There are many mountains and forests in this group. From Fig 8, the trip count of G1 are less than 1 per square kilometer, which means that this group is visited by few people using taxis. Another issue is that the trip count value in this group has more peaks in the afternoon than other groups. The extreme values for every day appeared from 12 pm to 5 pm. People usually travel for recreation at those times and seek leisure pursuits at these places until night. We can confirm that the main function of this group is the trip to the country.

Activities in G2 involve shopping, food, live, resident and transport as the main POIs. These POIs are also in the other groups, so it is difficult to distinguish one from another based on the type. We can discover, however, the function from the inflow curve shown in Fig 9. The inflow curve is a negative value from 6 am to 7 pm and is positive from 8 pm to 5 am. Of special interest is the fact that the value is approximately zero after 1 am. The inflow curve reflects that many people visit another region during working hours, and return after work. Meanwhile, it can be seen that the value in the morning is larger than in the evening, which means people like to take a vehicle for work in emergency circumstances but return home using other transportation. As a result, the main function is resident, based on the inflow curve analysis.

From the inflow curve, we see that G3 is also a mixture regions, similar to G2. The special feature of G3 is that it has business and accommodate in this group, with a ratio higher than that of G2 but lower than that of G4. This feature means G3 is a transitional region between G2 and G4. On the map, we can see they are mainly distributed around G2 and G4, and the areas around G1 and G2 are the suburban centers.

The trip count in G4 is much greater than in the previous group. The inflow curve is positive from 7 am to 11 am, and the peak time is at 9 am. It is the time period when people come to work. Thus, the characteristic means that many people work there. The shopping and business ratio is also the largest in all of the groups. It is definite that the main function of this group is a business area.

G5 and G6, are both special groups. There is only one region in these two groups. Trip count in G5 show that a large number of people take vehicles here starting at 10 am. The inflow can show us that it has positive value from 9 am to 4 pm, and negative value after 4 pm. Considering that there is an extremely large number of shops in this region, the group’s main function is shopping. The inflow curve of G6 is positive all day. From the POI ratio, we can find that there are many shops, food, and transport facilities here. It became evident that this region has a great deal of convenient transportation. In fact, we can see that G5 is a well-known shopping place, named Chunxi Road, and G6 is the Chengdu Railway Station.

As for the last group, G7 also has large number of people that visit there which can be seen from the trip count and inflow characteristics. From the POI ratio, we can see that the culture and shopping ratio are the predominant types. The inflow curve shows that the highest inflow value occurs after 11 am. The overall value of the curve increases sharply during the weekend. All of those factors indicate that G7 is a tourist group. In fact, the OSM shows some landmarks in this group such as the Temple of the Marquis, the Wide and Narrow Valley, etc. All of these locations contain sites of historical interest.

## 5. Conclusions

In this study, we developed a clustering method to help discover functional urban regions. This method applies GMM to classify regions’ inflow and trip count characteristics, and regroups urban regions using the PCC clustering method based on these typical characteristics. Using Chengdu’s vehicle trajectory data, we demonstrate how the method can differentiate between urban functional regions by comparing the proportion of surface objects in each region. This research shows that vehicle trajectory data in different functional urban regions has different time-series curves while similar types of functional regions can be identified by these curves.

There are some innovations that arose from this experiment. First, we found the series curves of inflow and trip count are a better means to represent the spatiotemporal patterns of residential travel than using pick-ups and drop-offs. Second, it has been proven that the method flow of GMM and PCC could identify different regions effectively. Finally, POIs could be taken into consideration when we define a region’s main function. There still is, however, some work that needs to be done in the future. Vehicle trajectory data is one way of recording residents’ daily travel but it can only reflect residents’ travel patterns to a certain extent. On the one hand, it is recommended that multi-source data combining with the buses, subway transportation, and mobile devices location data could be applied to better analyze residents’ travel patterns. On the other hand, with the development and growth of a city, the functional urban regions are changing over time, it is difficult to reflect the real distribution of functional urban regions in a timely and accurate fashion from official planning diagrams. On the contrary, the results of the clustering of urban function regions are easily obtained if the road network data, POI data, and track data were collected from different months or years, so that the change detection analyses of urban functional regions are accessible.
